# Antibodies Elicited in Response to a Single Cycle Glycoprotein D Deletion Viral Vaccine Candidate Bind C1q and Activate Complement Mediated Neutralization and Cytolysis

**DOI:** 10.3390/v13071284

**Published:** 2021-06-30

**Authors:** Maria Luisa Visciano, Aakash Mahant Mahant, Carl Pierce, Richard Hunte, Betsy C. Herold

**Affiliations:** 1 Departments of Microbiology and Immunology, Albert Einstein College of Medicine, Bronx, NY 10461, USA; maria.visciano@einsteinmed.org (M.L.V.); aakash.mahant@einsteinmed.org (A.M.M.); Carl.Pierce@einsteinmed.org (C.P.); richard.hunte@einsteinmed.org (R.H.); 2 Department of Pediatrics, Albert Einstein College of Medicine, Bronx, NY 10461, USA

**Keywords:** herpes simplex viruses, vaccines, complement, C1q, glycoprotein D, glycoprotein B, complement-dependent cytolysis, complement-dependent neutralization

## Abstract

Herpes simplex virus (HSV) prevention is a global health priority but, despite decades of research, there is no effective vaccine. Prior efforts focused on generating glycoprotein D (gD) neutralizing antibodies, but clinical trial outcomes were disappointing. The deletion of gD yields a single-cycle candidate vaccine (∆gD-2) that elicits high titer polyantigenic non-gD antibodies that exhibit little complement-independent neutralization but mediate antibody-dependent cellular cytotoxicity (ADCC) and phagocytosis (ADCP). Active or passive immunization with ΔgD-2 completely protects mice from lethal disease and latency following challenge with clinical isolates of either serotype. The current studies evaluated the role of complement in vaccine-elicited protection. The immune serum from the ΔgD-2 vaccinated mice exhibited significantly greater C1q binding compared to the serum from the gD protein vaccinated mice with infected cell lysates from either serotype as capture antigens. The C1q-binding antibodies recognized glycoprotein B. This resulted in significantly greater antibody-mediated complement-dependent cytolysis and neutralization. Notably, complete protection was preserved when the ΔgD-2 immune serum was passively transferred into C1q knockout mice, suggesting that ADCC and ADCP are sufficient in mice. We speculate that the polyfunctional responses elicited by ΔgD-2 may prove more effective in preventing HSV, compared to the more restrictive responses elicited by adjuvanted gD protein vaccines.

## 1. Introduction

Herpes simplex virus type 1 (HSV-1) and type 2 (HSV-2) are large DNA viruses that establish lifelong infection with periods of latency interspersed with episodes of asymptomatic or clinical reactivation. HSV-1 primarily causes oral mucocutaneous lesions, ocular disease and sporadic encephalitis, whereas both cause genital and neonatal infections. While HSV-2 dominates globally, HSV-1 is emerging as the more common cause of genital and neonatal disease in developed countries [1,2]. Immunocompromised hosts are at risk of more severe and prolonged symptoms with each of these syndromes and are at greater risk of viral dissemination. HSV-2, in particular, is a major cofactor fueling the HIV epidemic and is associated with an increased risk of HIV transmission and acquisition [3,4]. There are an estimated 3.7 billion people under the age of 50 years infected with HSV-1 and 491 million people between the ages of 15–49 years seropositive for HSV-2 [1,2], but despite the enormous health burden and the years of research in the field, there is no licensed HSV vaccine [5].

Various factors have contributed to the difficulty in designing an effective HSV vaccine, including the absence of defined correlates of immune protection and the ability of these viruses to evade innate and adaptive immune responses through diverse mechanisms [6]. All of the candidate vaccines that have been evaluated in clinical efficacy trials were predicated on the presumption that the neutralizing antibodies targeting the envelope glycoproteins D (gD) and/or B (gB) were correlates of immune protection [7,8,9,10,11]. However, results have been uniformly disappointing and highlight the need to consider other mechanisms of immune protection for vaccine development.

We recently engineered a single-cycle HSV-2 candidate vaccine strain deleted in gD (ΔgD-2), which provided complete protection against HSV-1 and HSV-2 and prevented the establishment of latency in murine models of vaginal, skin and ocular challenge [6,12,13,14,15,16,17]. The passive transfer of immune serum completely protected naïve mice from subsequent challenge, but surprisingly, there was little neutralization activity present. Rather, protection mapped to the ability of the Fc portion of the antibodies to engage Fc receptors and trigger antibody-dependent cellular cytotoxicity (ADCC), and to facilitate viral clearance by phagocytic cells [16,17]. The central role of the Fc receptor in mediating protection was confirmed by the observation that passive protection was lost when immune serum was transferred into FcγRIV knockout mice [18]. In contrast, adjuvanted recombinant gD protein vaccines elicited a predominant neutralizing response in the murine model, little ADCC and significantly less protection against disease following active or passive immunization when mice were challenged with clinical isolates. Moreover, the recombinant gD protein vaccine failed to prevent latency [15,18].

The Fc portion of antibodies may also protect through interactions with C1q. C1q binding may increase the potency of antibodies by modulating the stoichiometric requirements for neutralization [19], opsonization and phagocytosis, or activation of the complement cascade with the generation of membrane attack complexes on the infected cell surface, resulting in complement-dependent cytolysis (CDC). The prior neutralization studies were conducted after heat-inactivation of the ∆gD-2 immune serum and also did not evaluate C1q binding or CDC. Therefore, the current studies were designed to test the hypothesis that complement contributes to the immune protection mediated by ΔgD-2. We compared C1q binding, CDC and complement-dependent neutralization in vitro, using immune serum obtained from mice vaccinated with ΔgD-2 or an adjuvanted recombinant gD protein vaccine and then conducted passive transfer studies in C1q knockout mice.

## 2. Methods

### 2.1. Cells, Virus and Vaccines

Vero (African green monkey kidney, American Type Culture Collection (ATTC), Manassas, VA, USA), VD60 [20] and HaCat (human keratinocyte (ATCC PCS-200-011)) cells were grown in Dulbecco modified Eagle medium (DMEM) (Thermo-Fisher Scientific, Waltham, MA, USA) supplemented with 10% fetal bovine serum (FBS) (HyClone, Logan, UT) and 1% penicillin-streptomycin (Thermo-Fisher Scientific, Waltham, MA, USA). ΔgD-2 was propagated in VD60 cells and viral titers (PFU/mL) were quantified on complementing VD60 and non-complementing Vero cells [21]. HSV-1 (B^3^x1.1) [15], HSV-2(G) [22], HSV-2(333)ZAG [23] (which expresses a green fluorescence protein under control of the cytomegalovirus promoter inserted at an intergenic site) and HSV-2(4674) [24] were propagated on Vero cells. HSV-2 gD protein (gD-2) was synthesized by the Einstein Protein Core Facility and combined with 150 µg of alum (Imject-Thermo Scientific, Thermo-Fisher Scientific, Waltham, MA, USA) and 12.5 µg of monophosphoryl lipid A (MPL) (Invivogen, San Diego, CA, USA) (rgD-2/Alum-MPL) [15].

### 2.2. Murine Vaccinations

Female Balb/c, C57BL/6 and C1qa knockout mice (C1qa^tmld(EUCOMM)Wtsi^) were purchased from Jackson Laboratory (JAX, Bar Harbor, ME, USA). The use of mice for this study was approved by the Institutional Animal Care and Use Committee at the Albert Einstein College of Medicine, protocol 2018-0504. Balb/c or C57BL/6 mice (age 6 weeks) were vaccinated intramuscularly (prime) and then three weeks later (boost) with ΔgD-2, rgD-2/Alum-MPL or a control VD60 lysate, as previously described [12]. Blood was obtained by retro-orbital sampling one week and three weeks post-boost.

### 2.3. C1q Binding ELISA

Overnight at 4 °C, 96-well plates (96 Well Flat Bottom Immuno Plate MaxiSorp (Thermo Fisher Scientific, Waltham, MA, USA) were coated with either 450 ng/mL of HSV infected (HSV-1 B^3^x1.1 or HSV-2 G) or uninfected Vero cell lysates or with 250 ng/mL of HSV-1 recombinant gD-1 or gB-1 (produced in HEK293 cells by the Einstein Protein Core Facility), as described [18]. The plates were washed 4 times with PBS-0.5% Tween (washing buffer, WB) and then blocked with 5% Non-Fat Dry Milk (Bio-Rad, Hercules, CA, USA) in phosphate buffered-saline (PBS) with 0.1% Igepal-CA 630 (Millipore Sigma, St. Louis, MO, USA) (blocking buffer) for 1 h at room temperature (RT). The cells or proteins were then incubated (2 h RT) with serial two-fold dilutions of heat-inactivated immune sera followed by mouse C1q (1 µg/mL) (Complement Technology Inc., Tyler, TX, USA) and rat anti-mouse C1q-Biotin (0.5 µg/mL) (Cedarlane Labs, Burlington, NC, USA). Bound C1q was quantified using horse radish peroxidase conjugated streptavidin (Thermo Fisher Scientific, Waltham, MA, USA) (30 min, RT) followed by an addition of a substrate (OptEIA TMB Substrate Reagent Set, cat #555214, BD Bioscience, San Jose, CA, USA). The reaction was stopped by an addition of 2N H_2_S0_4_ and plates were read at 450 nm in a SpectraMax M5e Microplate Reader (Molecular Devices, San Jose, CA, USA). All dilutions were made in blocking buffer and plates were washed 4× with washing buffer after each step.

### 2.4. Viral Neutralization in the Absence or Presence of Complement

Neutralization was assessed using a plaque reduction assay with minor modifications [18]. Serial dilutions of heat-inactivated immune sera were incubated for 1 h at 37 °C with HSV-1 or HSV-2 (~100 PFU/well) in the absence or presence of 10% *v/v* rabbit complement (Cedarlane Labs, Burlington, NC, USA). Vero cells in 24-well cell culture plates (Costar-Corning, Kennebunk, ME, USA) were inoculated in duplicate with the virus–serum–complement mixture and plaques were counted after 48 h of incubation. The neutralization titer was defined as the highest dilution that yielded a 50% reduction in plaque numbers.

### 2.5. Complement Dependent Cytotoxicity

HaCat cells were infected with HSV-2(333)ZAG (0.1 PFU/cell) in 6-well cell culture plates (Costar-Corning, Kennebunk, ME, USA) for 1 h and then overlaid with media for 9 h. Cells were washed with PBS, and then heat-inactivated immune sera (1:50 dilution in incomplete DMEM) was added to cells. Plates were kept on ice for 30 min before adding 20% *v/v* rabbit complement. After a 4 h incubation at 37 °C in 5% CO_2_, cells were detached with 500 µL/well Accutase (Thermo Fisher Scientific, Waltham, MA, USA), washed twice with PBS and stained with Zombie-NIR (BioLegend, San Diego, CA, USA) for 20 min at RT. Stained cells were then fixed with 2% paraformaldehyde and read with a Cytek Aurora 5 Laser System Flow cytometer (Cytek Bioscience, Freemont, CA, USA). Data were analyzed with FlowJo Software, version 10 (FlowJo-BD, Franklin Lakes, NJ, USA).

### 2.6. Passive Transfer Studies

Pooled serum containing 750 µg of total IgG (quantified by IgG ELISA, Invitrogen, Carlsbad, CA) harvested from intramuscularly ΔgD-2 or control (VD60) vaccinated C57Bl/6 mice was inoculated intraperitoneally into naïve WT or C1q-knockout BL/6 mice 24 h prior to skin challenge with HSV-2(4674) (5 × 10 PFU/mouse [13] ), as previously described [18]. Mice were then monitored daily for epithelial and neurological disease and scored as follows: (1) erythema at inoculation site; (2) spread to distant site, zosteriform lesions, edema; (3) ulcerations, epidermal spread, limb paresis; (4) hind limb paralysis and (5) death. Mice were euthanized at a score of 4 and assigned a score of 5 the following day. At the time of euthanasia (when mice succumbed or day 14 post-challenge), sacral nerve tissue was collected and DNA isolated using the Qiagen Blood and Tissue DNA isolation kit (Qiagen, Hilden, Germany). A total of 10 ng of DNA per sample was loaded, and primers and probes specific for HSV polymerase were used to quantify HSV DNA (forward primer sequence, 5′-GGCCAGGCGCTTGTTGGTGTA-3′; reverse primer sequence, 5′-ATCACCGACCCGGAGAGGGA-3′; probe sequence, 5′-CCGCCGAACTGAGCAGACACCCGC-3′). Mouse β actin was used as a loading control (Applied Biosystems, Foster City, CA, USA), and qPCR was run in an Applied Biosystems QuantStudio 7 Flex.

### 2.7. Statistical Analysis

Analyses were performed using GraphPad Prism version 9 software (GraphPad Software, San Diego, CA, USA). The area under the curve (AUC) was calculated using trapezoid rule. A *p*-value of 0.05 was considered statistically significant. Survival curves were compared using the Gehan–Breslow–Wilcoxon test; other results were compared using *t*-tests or an ANOVA, as indicated.

## 3. Results

### 3.1. Immune Serum from ΔgD-2, but Not rgD-2Alum-MPL Vaccinated Mice Binds C1q

**Figure 1:**
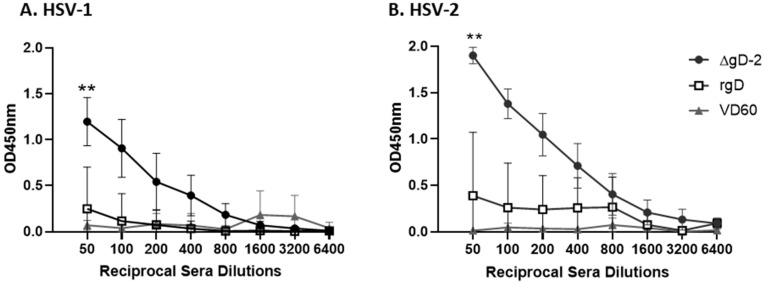
C1q binding of immune serum in mice vaccinated with ΔgD-2, adjuvanted recombinant gD or VD60 cell lysate. Serial 2-fold dilutions of heat-inactivated immune serum (1:50–1:6400) were assayed for ability to bind C1q using an ELISA with (**A**) HSV-1 B^3^x1.1-infected Vero cells or (**B**) HSV-2(G) infected Vero cells. Results are shown as mean ± standard deviation (SD of the optical density units (OD450 nm) at each dilution (*n* = 9–10 mice per group) and the area under the curve was compared to VD60 controls using unpaired *t*-test with Welch’s correction (** *p* < 0.01). There was no significant difference comparing C1q binding of adjuvanted rgD immune serum and VD60 controls.

C1q binds to antibodies that are bound to viral particles or virally infected cells to promote activation of the complement cascade leading to CDC, opsonization and phagocytosis and/or complement-dependent neutralization. To determine if antibodies elicited in response to ΔgD-2 or rgD-2/Alum-MPL vaccination in mice exhibiting complement binding activity, a C1q sandwich binding ELISA was performed with immune serum (*n* = 10 mice per group). The immune serum from the ΔgD-2 prime-boost vaccinated mice exhibited significantly more C1q binding compared to rgD-2/Alum-MPL or compared to the VD60-control vaccinated mice when either HSV-1 (Figure 1A) or HSV-2 (Figure 1B) infected cell lysates were used as the capture antigen (*p* < 0.001 for both). In contrast, there was no significant increase in the C1q binding of the immune serum from rgD-2/Alum-MPL compared to the control mice. Notably, there was greater C1q binding of the ΔgD-2 immune serum when HSV-2, compared to HSV-1, infected cell lysates were used as the capture antigen (unpaired *t*-test comparing area under curve (AUC), 1079.0 ± 531.3 vs. 641.2 ± 134.3, *p* = 0.02).

**Figure 2:**
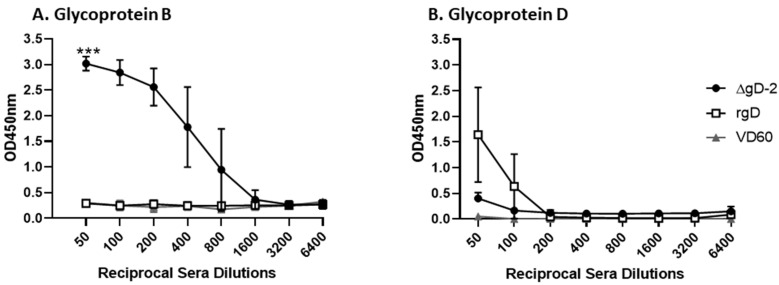
C1q binding of immune serum to glycoproteins gB or gD. Serial 2-fold dilutions of heat-inactivated immune sera (1:50–1:6400) were assayed for ability to bind C1q in ELISA with (**A**) recombinant gB or (**B**) recombinant gD protein. Results are shown as mean ± SD of the optical density units at each dilution (*n* = 10 mice per group) and the area under the curve was compared to VD60 controls by unpaired *t*-test with Welch’s correction (*** *p* < 0.001).

The ΔgD-2 vaccine elicits a polyantigenic response including antibodies that bind gB, whereas rgD-2/Alum-MPL elicits only gD-directed responses [17,18]. Since the envelope glycoproteins, which are also expressed on the surface of infected cells, are likely targets of C1q activating antibodies, we compared the C1q binding of immune serum to gB or gD. The immune serum from the ΔgD-2 prime-boost vaccinated mice exhibited significant C1q binding to gB (but not gD) compared to the control or rgD-2/Alum-MPL mice (*p* = 0.0003 comparing AUC, *n* = 10 mice/group) (Figure 2A). In contrast, there was no significant C1q binding of the rgD-2/Alum-MPL immune serum to gD (Figure 2B), which is consistent with a lack of significant C1q capture by infected cell lysates (unpaired *t*-test comparing AUC 700.4 ± 235 (rgD-2) vs. 609.8 ± 76 (VD60), *p* = 0.26).

### 3.2. gD-2 Immune Serum Mediates Complement Dependent Cytotoxicity

**Figure 3:**
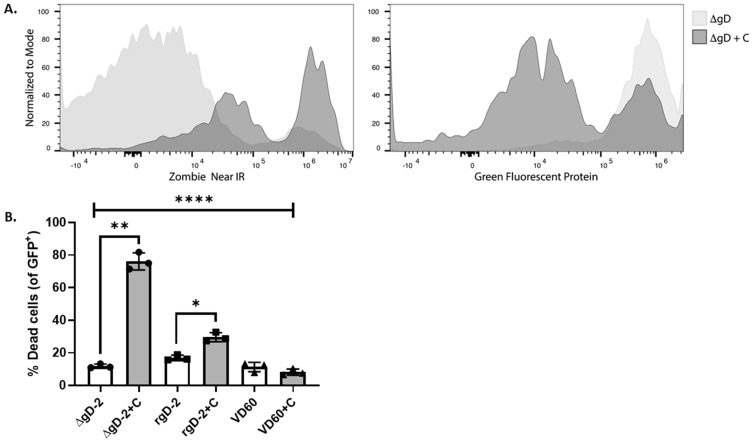
ΔgD-2 immune serum activates complement-dependent cytolysis. HaCAT cells were infected with HSV-2(333)ZAG, which expresses GFP, and then incubated for 4 h with heat-inactivated serum (1:50 dilution) obtained from mice prime-boost vaccinated with ΔgD-2, adjuvanted recombinant gD-2 (rgD-2), or VD60 control cell lysate with or without the addition of rabbit complement (+C). Cells were stained with Zombie NIR live-dead marker, fixed and then analyzed using flow cytometry to quantify the percentage of infected (GFP+) dead cells. (**A**) Representative histogram showing GFP staining (right) and Zombie NIR live/dead staining (left) when infected cells were incubated with ΔgD-2 immune serum obtained from a single mouse with or without complement. The *y*-axis is normalized to mode (e.g., the highest peak is set at 100% for each sample). (**B**) Bar graph showing percent of the GFP+ dead cells (mean ± SD) from a representative experiment (*n* =3 mice per group) (**** *p* < 0.0001, ** *p* < 0.01 and * *p* <0.05, paired *t*-test comparing cytolysis with or without complement and two-way ANOVA with Tukey’s corrections for multiple comparisons). Results are representative of 3 independent experiments.

One of the major effects of C1q capture by antibody-bound infected cells is the assembly of the terminal components of complement to form a membrane attack complex leading to cell lysis. To determine if C1q binding translated into complement dependent cytolysis (CDC), HaCat cells were infected with HSV-2(333ZAG) for 9 h before adding immune serum and rabbit complement, and then analyzed using flow cytometry. In pilot studies, we confirmed that gB and gD are expressed on the infected cell surface by 6 h post-infection. The percentage of infected (GFP+) cells that were killed by the ΔgD-2 immune serum increased significantly from a mean of 11.77 to 76.09% when complement was added (*p* < 0.001, paired *t*-test) and compared to the serum from the VD60-immunized mice, with (11.29%) or without (8.21%) complement (*p* < 0.0001, two-way ANOVA) (Figure 3A,B). The addition of complement to the rgD-2/Alum-MPL immune serum had modest but significant effects on cytolysis (16.84 without vs. 29.57% with complement, *p* = 0.04, paired-*t*-test) and compared to the serum from the VD60 immunized mice when complement was added (*p* < 0.05, two-way ANOVA). The CDC activity of the ΔgD-2 immune serum was significantly greater than observed with the rgD-2 or VD60 immune serum (*p* < 0.0001, two-way ANOVA). Notably, there was a reduction in the GFP expression when infected cells were incubated with the ΔgD-2 immune serum and complement, likely reflecting a release of intracellular GFP as the cell was killed (Figure 3A, right panel).

### 3.3. Complement Enhances Neutralization Potency of ΔgD-2 Immune Serum

**Figure 4:**
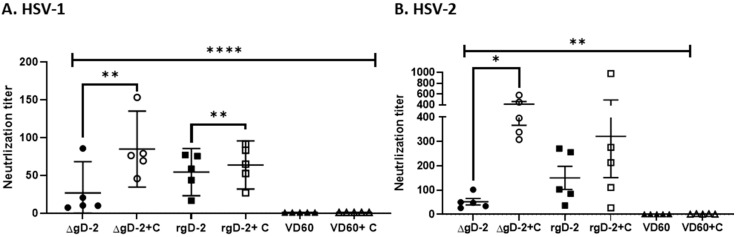
Complement enhances neutralization by ΔgD-2 immune serum. Neutralization of (**A**) HSV-1(B^3^x1.1) or (**B**) HSV-2 (G) infection was determined using a plaque assay with serial dilutions of heat-inactivated immune serum from ΔgD-2, adjuvanted recombinant gD protein (gD-2) or VD60 lysate-vaccinated mice in the absence or presence of 10% rabbit complement (C) compared to plaques formed in the absence of serum. The serum dilution that inhibits 50% of plaque formation is shown as mean ± sd (*n* = 5 mice). **** *p* < 0.0001, ** *p* < 0.01 and * *p* <0.05, paired *t*-test comparing neutralization titer with or without complement and two-way ANOVA with Tukey’s corrections for multiple comparisons.

To investigate whether complement augments viral neutralization, plaque reduction assays were performed with serial dilutions of heat-inactivated sera obtained from the ΔgD-2, rgD-2/Alum-MPL or control VD60 immunized mice in the absence or presence of 10% rabbit complement. The addition of complement increased the neutralization titer (concentration of serum that inhibited 50% of viral plaques) from 26.75 ± 33.2 to 84.67 ± 40.4 for ΔgD-2 against HSV-1(B^3^x1.1) (*p* = 0.0024, paired *t*-test) and from 52.3 ± 29.5 to 414.7 ± 107.1 for HSV-2(G) (*p* = 0.0025). The complement also increased the neutralization titer of the rgD-2 Alum/MPL immune serum against HSV-1 from 54.24 ± 25.08 to 63.75 ± 25/59 (*p* = 0.0055) but had no significant effect on the neutralization of HSV-2 (Figure 4). Notably, the complement-dependent neutralization titer of the ΔgD-2 immune serum was significantly higher than the complement-independent neutralization titer of the rgD-2/Alum-MPL2 immune serum for HSV-2 (*p* = 0.004, unpaired *t*-test).

### 3.4. C1q Is Not Required for Passive Protection

**Figure 5:**
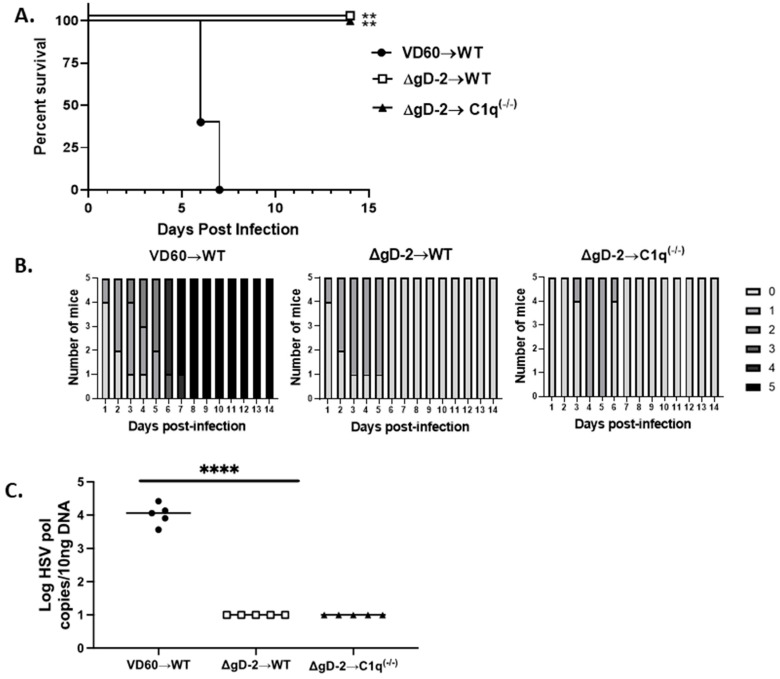
Passive transfer of immune serum from wild-type mice vaccinated with ΔgD-2 protects wild-type and *C1q-/-* mice. Pooled immune serum obtained from 5 ΔgD-2 vaccinated mice were assayed for IgG content and then administered intraperitoneally (750 μg total IgG/mouse) to wild-type or C1q-/- mice 1 day prior to challenging the mice by skin scarification with 5 × 10^4^ PFU/mice of HSV-2 (4674). Survival curves (**A**) and disease scores (**B**) are shown, and the survival was compared using the Gehan-Breslow-Wilcoxon test (*n* = 5 mice per group ** *p* < 0.01). (**C**) Viral spread to sacral ganglia was determined using a quantitative PCR at time of euthanasia, **** *p* < 0.0001, ANOVA.

Previous studies demonstrated that the immune serum from the ΔgD-2 vaccinated (but not the adjuvanted rgD-2 protein vaccinated) mice passively protected the wild-type but not FcγRIV knockout mice from viral challenge, indicating that the activation of this FcR is critical for immune protection [15,18]. To determine if C1q binding is also required for passive protection, serum from the ΔgD-2 vaccinated mice was pooled and an equivalent amount of total IgG (750 µg/mouse) was administered intraperitoneally 1 day prior to challenging naïve C57Bl/6 or C1q knockout mice on the skin with an LD90 dose of HSV-2(4674). As a control, the wild-type mice received 750 µg/mouse of immune serum from the mice that had been immunized with VD60 cell lysate. Although the mice developed mild epithelial signs within the first few days following challenge, both the wild-type and C1q knockout mice that received the ΔgD-2 immune serum had significantly lower disease scores (*p* < 0.0001) and completely recovered, whereas all of the controls succumbed (*p* < 0.01) (Figure 5A,B). Moreover, the passive transfer of the ΔgD-2 immune serum reduced the amount of HSV-2 DNA detected in sacral ganglia tissue using a PCR at the time of euthanasia to below the limit of detection (*p* < 0.0001 compared to VD60 controls) (Figure 5C).

## 4. Discussion

The current studies extend our prior observations, highlighting differences in the immune response to the ΔgD-2 and rgD-2/Alum-MPL vaccinations. In addition to the previously documented more potent ADCC activity [12,15,18], the antibodies elicited by ΔgD-2 exhibit greater C1q binding compared to those elicited by the adjuvanted recombinant gD protein vaccine and these antibodies are associated with enhanced complement-dependent cytolysis and neutralization against both serotypes. C1q plays a pivotal role in initiating the lysis of virally infected cells by activating the classical complement cascade, which results in the assembly of the membrane attack complex on the cell surface and the formation of pores that disrupt the cell membrane. The mechanisms by which complement enhances antibody-dependent neutralization are not fully understood but may be mediated by C1q binding to the Fc component, thereby reducing the number of IgG molecules needed to neutralize viral particles, as was demonstrated for the West Nile virus [19]. Others have suggested that, in addition to C1q, the recruitment of other complement components (C4, C2 and C3) to the viral surface sterically blocks interactions between the virus and its receptors to prevent viral entry, as originally observed in studies with the Epstein–Barr virus [25].

Our prior findings, that antibodies elicited by the ΔgD-2 vaccine were only weakly neutralizing, were conducted with heat-inactivated serum (complement-independent) but were somewhat surprising, as the vaccine elicits polyantigenic responses that target glycoproteins B, C and E as well as other non-envelope viral proteins [17]. While gD is the dominant target of neutralizing responses, gB is also a major target [26]. However, preclinical rabbit studies of cytomegalovirus (CMV) gB vaccines found little neutralizing activity in the absence of complement. The addition of complement increased the neutralization titer, and a similar result was observed when complement was added to the human immune serum obtained from the participants in the CMV gB/MF59 clinical vaccine trials [27]. Monoclonal antibody (mAb) studies suggested that the epitope targeted, rather than the antibody avidity, determined the ability of the CMV gB mAbs to mediate complement-dependent neutralization. Our findings here suggest a similar phenomenon for ΔgD-2. Presumably, the stoichiometry of gB antibodies elicited by the ΔgD-2 vaccine and the epitopes targeted limit neutralization in the absence of complement.

The addition of complement to the neutralization assays had a greater effect for the ΔgD-2 immune serum, particularly against HSV-2, compared to the rgD-2 immune serum. The limited effect of complement on the rgD-2/Alum-MPL immune serum likely reflects the immune evasion properties of gE and gC. Specifically, gE binds to the Fc component of immunoglobulins to inhibit C1q binding, whereas gC binds C3b to inhibit complement-mediated neutralization and cytolysis. The deletion of gC from HSV-1 or HSV-2 results in an increased neutralization activity in the presence of human serum [28] and the neutralization titer of HSV gB mAbs was increased 2–16-fold when assayed against a gC null virus [28]. The importance of the antibodies that block the gE and gC immune evasion strategies is further supported by preclinical studies with a trivalent subunit protein or mRNA vaccine comprised of gD, gC and gE, which showed increased neutralizing titers and greater protection in small animal models compared to gD alone [29,30]. Presumably, ΔgD-2 overcomes these immune evasion strategies because it generates antibodies to gC and gE. This likely contributes to the observation that the neutralization titer of the ΔgD-2 immune serum in the presence of complement was at least as great as the neutralization titer of the rgD-2/Alum-MPL immune serum in the absence or presence of complement.

Although our in vitro studies demonstrated a role for complement in enhancing the neutralization and cytolytic activity of the ΔgD-2 immune serum, complete protection was preserved in passive transfer studies into C1q knockout mice, suggesting that the ADCC activity is sufficient to mediate protection in the murine model. This does not preclude the possibility that complement binding will contribute to protection in humans as the weaker activity of intrinsic mouse complement compared to humans may underestimate its role in mediating immune protection [31].

In summary, the current studies expand the characterization of the functionality of antibodies elicited in response to ΔgD-2 immunization, which includes not only the previously identified ADCC and antibody-dependent cellular phagocytosis (ADCP), but also antibody-mediated complement-dependent neutralization and cytolysis. This contrasts with recombinant gD protein vaccines, which elicit an almost exclusive complement-independent neutralization response. This more restrictive functional antibody response failed to protect in clinical trials [8]. Polyfunctional antibody responses are likely to be important for the prevention and control of other viral infections, including, for example, HIV, Ebolavirus and SARS-CoV-2 [32,33,34,35]. We speculate that prophylactic and therapeutic vaccines that elicit polyfunctional responses will prove more effective in preventing HSV and other viral infections.

## Data Availability

Data is available upon request.
